# Type 1 Diabetes Mellitus and Autoimmune Diseases: A Critical Review of the Association and the Application of Personalized Medicine

**DOI:** 10.3390/jpm13030422

**Published:** 2023-02-26

**Authors:** Mihaela Simona Popoviciu, Nirja Kaka, Yashendra Sethi, Neil Patel, Hitesh Chopra, Simona Cavalu

**Affiliations:** 1 Faculty of Medicine and Pharmacy, University of Oradea, 410087 Oradea, Romania; 2 PearResearch, Dehradun 248001, India; 3 Department of Medicine, GMERS Medical College, Himmatnagar 383001, India; 4 Department of Medicine, Government Doon Medical College, HNB Uttarakhand Medical Education University, Dehradun 248001, India; 5 Chitkara College of Pharmacy, Chitkara University, Rajpura 140401, India

**Keywords:** diabetes, T1DM, autoimmune diseases, autoimmunity

## Abstract

Type 1 Diabetes Mellitus (T1DM) is a common hyperglycemic disease characterized by the autoimmune destruction of insulin-producing beta cells of the pancreas. Various attempts have been made to understand the complex interplay of genetic and environmental factors which lead to the development of the autoimmune response in an individual. T1DM is frequently associated with other autoimmune illnesses, the most common being autoimmune thyroid disorders affecting more than 90% of people with T1D and autoimmune disorders. Antithyroid antibodies are present in around 20% of children with T1D at the start of the illness and are more frequent in girls. Patients with T1DM often have various other co-existing multi-system autoimmune disorders including but not limited to thyroid diseases, parathyroid diseases, celiac disease, vitiligo, gastritis, skin diseases, and rheumatic diseases. It is a consistent observation in clinics that T1DM patients have other autoimmune disorders which in turn affect their prognosis. Concomitant autoimmune illness might affect diabetes care and manifest itself clinically in a variety of ways. A thorough understanding of the complex pathogenesis of this modern-day epidemic and its association with other autoimmune disorders has been attempted in this review in order to delineate the measures to prevent the development of these conditions and limit the morbidity of the afflicted individuals as well. The measures including antibody screening in susceptible individuals, early identification and management of other autoimmune disorders, and adoption of personalized medicine can significantly enhance the quality of life of these patients. Personalized medicine has recently gained favor in the scientific, medical, and public domains, and is frequently heralded as the future paradigm of healthcare delivery. With the evolution of the ‘omics’, the individualization of therapy is not only closer to reality but also the need of the hour.

## 1. Introduction

Type 1 Diabetes Mellitus (T1DM) is a chronic disease characterized by the inability of the body to produce insulin due to the autoimmune destruction of the beta cells in the pancreas. Insulin is a key anabolic hormone that has numerous effects on glucose, lipid, protein, and mineral metabolisms in addition to growth. T1DM thus presents as a systemic disease characterized by the phenotype of hyperglycemia. Numerous studies have demonstrated that genetic factors contribute significantly to the development of type 1 diabetes. The major susceptibility gene is located in the HLA region of chromosome 6, with a strong link to alleles DR3, DR4, DQA1∗0501, DQB1∗0201, DQA1∗0301, and DQB1∗0302. Approximately a 40–50% risk of developing T1DM is attributed to the HLA complex [1].

Recent studies indicate a significant global increase in the prevalence of type 1 diabetes. The prevalence also varies globally from 3.5:10,000 in Africa to 12.2:10,000 in the United States of America. In the period 1989–2008, an increase in the incidence of approximately 3–4% per year was observed in Europe [2].

Various environmental factors—such as viral infections, cow’s milk proteins, and vitamin D3 deficiency—have been implicated as triggers to the autoimmune process in genetically susceptible individuals, but none of them have been conclusively linked to diabetes [3]. Immunological markers of T1DM include anti-pancreatic islet cell antibodies, anti-glutamate decarboxylase (GAD) antibodies, anti-insulin antibodies, anti-tyrosine phosphatase antibodies, and anti-zinc transporter 8 antibodies [4].

The autoimmune process that induces T1DM can also affect other organs, leading to the development of additional autoimmune diseases and complicating diabetes management. T1DM is most frequently associated with autoimmune thyroid diseases (Hashimoto’s thyroiditis and Graves’ disease) in a percentage of 17–30%, Addison’s disease at 0.2%, celiac disease at 8%, autoimmune gastritis at 5–10%, but with rheumatoid arthritis at 1.2% or systemic lupus erythematosus at 1.15% [5,6,7,8,9].

T1DM is an autoimmune disease characterized by an autoimmune response against pancreatic beta cells. T1DM is frequently associated with other autoimmune illnesses, and anti-islet autoantibodies appear before the clinical disease manifests. These comorbid autoimmune diseases can affect the natural history of the disease largely, warranting due consideration. The review was done to comprehensively and critically compile all evidence of the association between autoimmune diseases and T1DM; the authors also underline the need for inclusive and comprehensive treatment of the patient as a whole rather than only treating the disease—T1DM.

## 2. Materials and Methods

A literature search was conducted on electronic databases of PubMed, Web of Science, EMBASE, and Cochrane library to identify and critically review the association of autoimmune diseases (AID) with type 1 diabetes. 

## 3. T1DMand Autoimmune Endocrine Pathology

### 3.1. Hashimoto’s Thyroiditis and Graves’ Disease

The HANES III study reported a higher prevalence of thyroid disease in patients diagnosed with diabetes compared to the general population [10]. Autoimmune thyroid diseases occur in 17% to 30% of patients with type 1 diabetes. The close relationship between these conditions is largely explained by the sharing of a common genetic background. HLA antigens DQ2 (DQA1∗0501-DQB1∗0201) and DQ8 (DQA1∗0301-DQB1∗0302), closely related to DR3 and DR4, are the common predisposing factors [5].

Autoimmune thyroid diseases associated with diabetes include Hashimoto’s chronic autoimmune thyroiditis and Basedow–Graves’ disease. Hashimoto’s thyroiditis is characterized by the presence of antithyroperoxidase antibodies and antithyroglobulin antibodies. Hashimoto’s thyroiditis is associated with normofunction, thyroid hyperfunction, or thyroid hypofunction, the latter being the most common form. Basedow’s disease is characterized by the presence of antibodies against thyrotropin receptors (TRAb), which cause an increase in thyroid volume and hyperthyroidism [11]. These antibodies are detected only in 17–25% of patients at the time of diagnosis with type 1 diabetes; in most cases, they appear during the evolution of diabetes, 2–3 years after the diagnosis [12].

The prevalence of clinical hypothyroidism in patients with T1DM varies between 4 and 18% compared to the general population (5–10%). The prevalence of hyperthyroidism due to Graves’ disease or the hyperthyroid phase of Hashimoto’s thyroiditis in diabetic patients is lower than that of hypothyroidism (1.5–4%). The prevalence of both conditions—hypothyroidism and hyperthyroidism—is higher than in the non-diabetic population [13,14,15]. Thyroid hormones exert profound effects on carbohydrate and lipid metabolism. Thyroid hormone receptors play an important role in the normal development of pancreatic islets [14,15]. 

Neonatal β-cells have receptors for thyroid hormones, and their exposure to the T3 hormone (triiodothyronine) determines β-cell maturation and stimulates insulin secretion. In addition, T3 promotes the proliferation of pancreatic islet cells [15]. Thyroid hormones increase hepatic glucose production by increasing hepatic expression of the glucose transporter GLUT 2 and stimulate endogenous glucose production by increasing gluconeogenesis and glycogenolysis processes. This decreases the insulin sensitivity of hepatocytes [16]. Both lipogenesis and lipolysis are stimulated by T3 and T4. Lipolysis is largely mediated through the increase in the number of hepatic receptors for low molecular density lipoproteins (LDL) and accelerating LDL clearance [17]. Therefore, thyroid dysfunction can increase cardiovascular risk due to the interaction between dyslipidemia, increased peripheral resistance to insulin, and vascular dysfunction [18].

Hyperthyroidism is characterized by a hypermetabolic state with increased energy consumption, decreased cholesterol levels, increased lipolysis, and enhanced gluconeogenesis [19]. Patients with hyperthyroidism may be at increased risk of severe hyperglycemia, and pre-existing diabetes is exacerbated by hyperthyroidism [20].

Hyperthyroidism is a promoter of the hyperglycemic state. Due to an accelerated rate of degradation and a significant release of physiologically inactive insulin precursors, hyperthyroidism reduces the half-life of insulin [21]. In uncontrolled Graves’ disease, proinsulin levels in response to food intake are elevated [22].

Another mechanism that explains the relationship between hyperthyroidism and hyperglycemia is the increased intestinal absorption of glucose mediated by the excess of thyroid hormones [4]. Thyroid hormone increases GLUT2 concentrations in the plasma membrane of hepatocytes. In addition, an increase in lipolysis is observed in hyperthyroidism. Diabetic patients with hyperthyroidism experience worsened glycemic control. Thyrotoxicosis precipitating ketoacidosis has been demonstrated [16]. Patients with Basedow–Graves’ disease who have Graves’ ophthalmopathy and diabetes have a higher risk of optic neuropathy, which can lead to blindness [23].

The treatment of hyperthyroidism with synthetic antithyroid drugs does not directly affect the blood glucose level. Corticosteroids are occasionally used for the treatment of Graves’ ophthalmopathy, and, due to the negative effects on metabolic control, they should be administered with caution to diabetic patients with Graves’ disease. Patients with diabetes who develop hyperthyroidism should be considered for a modification of their insulin therapy. Patients experiencing symptoms and clinical evidence of ketoacidosis should have their thyroid function assessed [5]. Hypothyroidism is characterized by decreased intestinal absorption of glucose and reduced hepatic and muscular gluconeogenesis and glycogenolysis [4]. The dysregulation of leptin action at the hypothalamus level, impairment of GLUT4 translocation, and elevation of free fatty acids are some known contributing factors to the pathogenetic process that underlies insulin resistance in hypothyroidism [19].

Hypothyroidism in diabetic patients is successfully treated by levothyroxine monotherapy. Excessive levothyroxine (LT4) therapy-induced TSH suppression should be avoided because it can induce iatrogenic hyperthyroidism and cause an additional deterioration of glycemic metabolism. In individuals with hypothyroidism, LT4 replacement treatment largely normalizes the lipid profile but combination therapy with statins is frequently required to achieve improved lipid profile management [24].

Recurrent episodes of hypoglycemia are clues to the development of hypothyroidism, and thyroid hormone replacement reduces blood glucose fluctuations in diabetic patients. Patients with hypothyroidism require lower doses of insulin due to the low production of hepatic glucose, and when the need for exogenous insulin is not adapted, symptomatic hypoglycemia ensues [25].

The association between T1DM and autoimmune thyroid diseases is known as autoimmune polyglandular syndrome type III and is the most common subtype of autoimmune glandular diseases. Autoimmune polyglandular syndromes (PAS) represent associations of at least two autoimmune-mediated endocrine disorders. PAS type I includes chronic mucocutaneous candidiasis, hypoparathyroidism, and Addison’s disease. PAS type II includes T1DM, Addison’s disease, and at least one other autoimmune endocrinopathy (Hasimoto’s thyroiditis or Graves’ disease); PAS type III includes autoimmune thyroid disease in combination with T1DM, but excluding Addison’s disease; PAS type IV is a combination of at least two autoimmune endocrinopathies but excluding types I–III [26].

### 3.2. Addison’s Disease

Addison’s disease is a rare endocrine disorder characterized by an insufficient synthesis of the steroid hormones cortisol and aldosterone by the two outer layers of adrenal gland cells (adrenal cortex), resulting in adrenal insufficiency. Chronic adrenal insufficiency or Addison’s disease is due to autoimmune etiology in 80% of cases. It can be present as an isolated autoimmune adrenal deficiency or as part of the aforementioned autoimmune polyglandular syndromes: PAS type I or type II. PAS type I results from the mutation of the AIRE gene located on chromosome 22q22.3. PAS type I is unrelated to HLA genes. The most frequent association of Addison’s disease is within PAS II, having HLA-DR3 or HLA-DR4 or both as a genetic substrate. The main autoantibodies involved in triggering adrenal autoimmunity are anti-21-hydroxylase antibodies [27].

The simultaneous existence of Addison’s disease and T1DM is rare; up to 14% of patients with Addison’s disease have associated T1DM, while the prevalence of autoimmune Addison’s disease among patients with T1DM is 0.2% [6]. Diabetes precedes the development of adrenal insufficiency in most patients and often occurs at a young age [28]. The simultaneous association of the two diseases leads to frequent episodes of hypoglycemia due to the decrease in gluconeogenesis and the increased sensitivity to insulin. Thus, adrenal insufficiency due to autoimmune causes should be considered in the case of patients with T1DM who suffer from unexplained recurrent hypoglycemia and fatigue [26].

Insulin and glucocorticoid replacement therapies often raise difficulties. They have opposite effects on glucose metabolism, with insulin decreasing and glucocorticoids increasing the plasma glucose level. Glucocorticoids induce insulin resistance and lower glucose tolerance in susceptible individuals. In the absence of physiological levels of circulating glucocorticoids, insulin sensitivity is increased, leading to increased peripheral glucose utilization, reduced gluconeogenesis, and hepatic glucose release. The latter could be the reason patients with T1DM require a lower dose of insulin at the time of Addison’s disease. The incorrect association of the two therapies predisposes patients to the risk of hypo- or hyperglycemia, diabetic ketoacidosis or Addisonian crisis [29]. Elbelt et al. (2009) studied insulin requirements in patients with T1DM and Addison’s disease treated with oral hydrocortisone or cortisone acetate divided into two or three doses per day, using either continuous subcutaneous insulin administration or conventional insulin injection therapy. It was observed that patients suffering from both autoimmune diseases compared to those suffering only from T1DM have a lower need for basal insulin and an increased need for post-prandial insulin [30]. 

### 3.3. Autoimmune Hypoparathyroidism

Hypoparathyroidism is a prominent component of autoimmune polyglandular syndrome type I. More than 80% of patients with PAS type I have hypoparathyroidism, which may be their only endocrinopathy [31]. T1DM is found in 1–18% of patients with PAS type I and appears earlier, at an age younger than 21 years [32]. NALP5 (NACHT leucine-rich-repeat protein 5) expression is up-regulated in parathyroid cells and associated with increasing calcium concentrations. This was suggested to be involved in the process of calcium-sensing or homeostasis in parathyroid chief cells [33].

Several studies have shown that screening for T1DM in patients diagnosed with PAS I is less effective. They showed that not all patients with PAS I who present antibodies against pancreatic islet cells develop diabetes [32].

### 3.4. T1DM and Celiac Disease

Celiac disease is a chronic autoimmune condition of the small intestine that is characterized by the destruction of the intestinal mucosa through an immune mechanism, occurring on a predisposing genetic background in response to exposure to gluten [34]. Celiac disease is one of the most common autoimmune conditions associated with type 1 diabetes. The prevalence of celiac disease in patients withT1DM is approximately 8%, significantly higher compared to the prevalence of celiac disease in the general population, estimated at 1% [7]. In over 90% of cases, the diagnosis of T1DM precedes that of celiac disease [35]. The occurrence of celiac disease seems to be significantly higher in children diagnosed with T1DM at young ages, under four years. Moreover, the risk of developing both conditions is higher for females than for males [36].

The mechanism of association of these two diseases involves a common genetic background: the hyperexpression of HLA-DQ2 and HLA-DQ8 antigens, which predispose individuals to the development of both diseases. The risk of developing celiac disease in patients with diabetes is partially increased by the presence of HLA DQA1∗0501, DQB1∗0201 [37].

The studies also showed similar environmental factors. The introduction of gluten into the infant’s diet before the first four months of life or after seven months has increased the risk of diabetes. Norris et al. (2003) hypothesized that celiac disease facilitates diabetes through the intestinal permeability mediated by inflammation by gluten [38]. It has been shown that an early diagnosis of celiac disease and the removal of gluten from the diet reduces the prevalence of associated autoimmune diseases, including type 1 diabetes, by reducing the duration of exposure to gluten [7].

Another environmental factor that plays a role in the pathogenesis of both diseases is viral infections. There are data that implicate enteroviruses as a specific risk factor in the onset of T1DM, and rotavirus infection in childhood as a risk factor for the onset of celiac disease [39,40].

In addition, vaccination against rotavirus had a protective effect in children exposed to gluten before the age of 6 months, indicating a possible interaction between diet and infections regarding the risk of autoimmune pathology [41]. A quarter of cases of celiac disease associated with T1DM are asymptomatic. Gastrointestinal manifestations of celiac disease include diarrhea, abdominal pain, flatulence, malnutrition, and constipation. Among the most frequent extraintestinal manifestations are short stature and iron deficiency anemia, present in approximately 50% of cases [42]. Other rare extraintestinal manifestations that suggest the presence of celiac disease in diabetic patients are weight loss, osteopenia, delayed puberty, and hypoglycemic episodes [43]. Hypoglycemia would be explained by the altered absorption of carbohydrates from the intestinal level [44]. Both intestinal and extraintestinal manifestations occur more frequently in children than in adults [45]. Serological testing is an important method of detecting celiac disease, especially in the case of diabetic patients, many of whom may be asymptomatic. In the case of positive serology, with a serological marker with a specificity of 100% to confirm the diagnosis, it is necessary to perform a duodenal biopsy, the ‘gold standard’, for the diagnosis of gluten enteropathy. IgA anti-tissue transglutaminase antibodies show the highest sensitivity, allowing the identification of approximately 98% of cases of celiac disease in patients with T1DM and representing the first-line analysis in identifying the condition. Although less sensitive (95%), anti-endomysium antibodies show a specificity of 99%. Antigliadin antibodies were used in the screening of celiac disease but are no longer recommended due to the lower sensitivity and specificity than the other classes of antibodies. It needs to be emphasized that the tests must be performed while the patient is following a gluten-containing diet to avoid false negative results. The serological screening of celiac disease must be performed in diabetic patients at different times throughout the disease course. It has been reported that screening at the onset of T1DM allows the identification of 1% of patients with celiac disease and that this prevalence increases to at least 5% in the following 5 years [45]. According to other research, antibody positivity can occur within 10 years from the initial diagnosis of T1DM and, in 90% of cases, within the first 2 years from diagnosis. Therefore, if serological tests for gluten enteropathy are negative at the onset of diabetes, patients must be reevaluated annually in the first 4 years and once every 2 years in the following 6 years [3].

The exclusion of gluten from the diet of diabetic patients diagnosed with celiac disease normalizes the architecture of the mucosa of the small intestine, allowing a normal absorption of nutrients and improving symptoms without greatly influencing the evolution of diabetes [7]. Several studies have shown that diabetic patients with untreated celiac disease have a lower body mass index and lower glycosylated hemoglobin values compared to diabetics without celiac disease. Untreated celiac disease and subsequent weight loss can improve glycemic control, but adherence to a gluten-free diet increases nutritional absorption from the intestinal level and consequently increases the need for insulin. It has been shown that the gluten-free diet decreases the number of hypoglycemic episodes, but the level of glycosylated hemoglobin remains relatively unchanged [7]. The assessment of the glycemic index of gluten-free foods remains a disputed subject. There are studies showing that gluten-free foods have a higher glycemic index than similar products that contain gluten, a fact explained by the different ingredients and procedures used in the manufacture of gluten-free products [46].

According to other studies, the introduction of gluten-free foods into the diet of diabetic patients does not compromise glycemic control, indicating that there are no differences between the glycemic index of gluten-free products and those containing gluten [47]. The improvement of intestinal absorption and the high consumption of gluten-free commercial foods, particularly those rich in carbohydrates and lipids, can favor the appearance of the metabolic syndrome, with an increase in the risk of cardiovascular disease even more than the isolated existence of type 1 diabetes. On the other hand, compliance with such a diet has the long-term benefits of avoiding the complications of celiac disease: malnutrition, osteoporosis, intestinal lymphoma, intestinal adenocarcinoma, etc. [48].

### 3.5. Autoimmune Gastritis

Autoimmune gastritis is characterized by atrophy of the gastric body and fundus and by the presence of antibodies against parietal cells and intrinsic factor. Antibodies against gastric parietal cells were detected in 60–85% of patients with chronic autoimmune gastritis, and antibodies against intrinsic factors in 30–50% of patients. The prevalence of autoimmune gastritis in the general population is 1–2%, increasing with age. In patients with type 1 diabetes, the prevalence is 5–10% [8].

Antibodies against parietal cells, the main immunological markers, react against the H^+^/K^+^ ATP-ase from the gastric wall, and the chronic attack on the proton pump leads to a decrease in gastric acid secretion. Antibodies against intrinsic factors cause vitamin B12 deficiency, which ultimately leads to pernicious anemia. Another characteristic marker of autoimmune gastritis, the low serum level of pepsinogen I, resulting from the destruction of the main cells, was identified as an early marker of pernicious anemia in patients with type I diabetes [49]. Anti-parietal cell antibodies predicted the occurrence of chronic atrophic gastritis in a prospective study conducted over a period of 5 years and with 208 adult patients with autoimmune thyroid disease. Antibody levels increased progressively over time, reached a peak, and then decreased, with the loss of the thyroid antigenic target and the progressive destruction of the gastric mucosa being observed [50]. Anti-parietal cell antibodies and low pepsinogen were parameters used to identify patients with a higher risk of cobalamin deficiency in a prospective study conducted over a period of 5 years and with 186 patients with T1DM [51]. These studies suggest that in addition to being markers of autoimmune gastritis, antibodies against parietal cells also predict gastric atrophy and its hematological manifestations [52]. Autoimmune gastritis is also characterized by iron deficiency anemia and hypergastrinemia. Although the absorption of iron does not occur/happen in the stomach, the decrease in gastric acidity influences the intestinal absorption of iron and can lead to the appearance of iron deficiency anemia [53].

The greatly increased serum gastrin is subsequent to G cell hyperplasia in the antrum. Hypergastrinemia causes hyperplasia of enterochromaffin-like (ECL) cells, which can progress to dysplasia and, finally, to the development of carcinoid tumors and gastric cancer [54].

In the case of patients with a high titer of anti-parietal cell antibodies and hypergastrinemia, upper digestive endoscopy and multiple biopsies should be performed. Endoscopically, the mucosa of the gastric body and fundus has atrophic characteristics: pale, with erased folds and a visible venous pattern. Biopsy specimens highlight lymphocytic infiltrate at the level of the submucosa, the marked reduction in the number of parietal cells and main cells, and, in some situations, even the appearance of intestinal metaplasia [55]. The involvement of *Helicobacter pylori* infection in the occurrence of autoimmune gastritis is controversial. Multiple studies support the involvement of infection, reporting an increased prevalence of seropositivity for *H. pylori* in patients with chronic autoimmune atrophic gastritis. On the other hand, other studies observed no link or only a negative link between *H. pylori* and autoimmune gastritis [52]. The presence of the specific HLA haplotype—HLA-DQA1∗0501-DQB1∗0301 in patients with T1DM increases the risk of autoimmune gastritis. This genotype is also associated with the presence of anti-glutamate decarboxylase (GAD) and ATPO antibodies. This correlation was observed both in the adult population and in children with T1DM [56].

A possible hypothesis suggests that the pancreas, thyroid, and stomach have common autoantigens. The GAD antigen is present in the pancreas, as well as in the gastric mucosa and submucosa, where it is involved in the conversion process of glutamic acid into gamma-aminobutyric acid, promoting the production of hydrochloric acid by the parietal cells. It is possible that the response of T cells to the GAD antigen begins in the pancreas, and then the autoimmune process expands, with active T cells initiating the process of destruction of other neuroendocrine tissues [3]. Early detection of autoimmune gastritis is important for the prevention and treatment of iron deficiency and pernicious anemia, as well as precancerous and cancerous gastric lesions. The level of anti-parietal cell antibodies should be tested at the onset of diabetes, then annually for 3 years, and then once every 5 years, or at any other time if there are clinical indications. Serological screening should be performed especially in patients with positive anti-glutamate decarboxylase and ATPO antibodies. It is also recommended that they perform a complete blood count annually and receive a dosage of serum gastrin, sideremia, ferritin, and vitamin B12 [56].

### 3.6. T1DMand Autoimmune Skin Pathology

Diabetes mellitus is a multifactorial endocrine disease with a severe global health impact. Hyperglycemia causes damage to a multitude of cell types, which not only includes the endothelial cells, neurons, and kidney cells but also keratinocytes and fibroblasts. Skin-related complications can be found in approximately one-third of all people diagnosed with diabetes and frequently appears before diagnosis, thus playing an important role in the recognition of the disease [57].

Almost all diabetic patients eventually develop skin complications following the long-term effects of diabetes on microcirculation and skin collagen. Skin infections are more common in type 2 diabetes, while autoimmune lesions are more common in T1DM [58]. To understand the development of skin lesions and their relationship with diabetes complications, a useful approach would be a long-term follow-up of type 1 DM patients and/or investigations of skin conditions in younger diabetic patients. Different skin manifestations, some specific and others non-specific (related to metabolic changes), can be observed in patients with diabetes. These include recurrent fungal or bacterial skin infections, lipoid necrobiosis, diabetic blisters, or autoimmune skin disorders. Diabetic dermopathy is the most common dermatological condition that can occur in up to 70% of adult patients with diabetes [59]. Although it is known that diabetes is associated with a series of skin manifestations, there are relatively few studies that analyze the prevalence of skin changes in young patients with type 1 diabetes. Skin manifestations generally appear after the development of diabetes, but they can also be the first sign or even precede the diagnosis by many years [60]. Next, we will review some aspects related to the association of T1DM with certain autoimmune skin diseases.

#### 3.6.1. Hives

Urticaria is an inflammatory skin condition that affects up to 20% of the world’s population at some point in life. This is described by the presence of plaque papules and/or angioedema due to the activation and degranulation of skin mast cells and the release of histamine and other chemical mediators. Most cases of urticaria are acute urticaria, which lasts less than 6 weeks and can be associated with infections or consumption of medicines or food. Chronic urticaria lasts longer than 6 weeks and persists for more than a year in most patients. Chronic urticaria affects the patient’s quality of life and is linked to psychiatric comorbidities and the high costs of medical care [61]. Chronic urticaria is a common disease in which most cases were considered idiopathic. Recent evidence indicates that some cases of chronic urticaria are of autoimmune origin [62]. The autoimmune subgroup is implicated in the α subunit of the IgG anti-Ig-E receptor in 35–40% of patients and with IgG anti-Ig-E in 5–10% of cases. It has been reported that these autoantibodies activate basophils and skin mast cells in vitro with increased release of C5a. The activation of basophils or mast cells that causes the release of histamine is characteristic of the autoimmune pathomechanism. Autoimmune pathologies such as thyroid disease and T1DM increase the chances of urticaria, and, therefore, it is believed that almost 45% of diagnosed patients have chronic autoimmune urticaria and the rest are idiopathic [63]. Confino-Cohen and colleagues reported the association of chronic urticaria with chronic autoimmune thyroiditis and the presence of antithyroid antibodies, with rheumatoid arthritis, Sjogren’s syndrome, celiac disease, type 1 diabetes, systemic lupus erythematosus, and others [62]. A study conducted between 1998 and 2011 in Taiwan looked at the association between type 1 DM and urticaria. The purpose of this study was to observe if children with T1DM have a higher or lower incidence of hives. A total of 5895 participants (1179 patients in the experimental group and 4716 in the control group) were taken for the study. Approximately 53% of the participants were female and the total incidence rate of urticaria in T1DM patients was 26.6 per 1000 people, and in the control group it was 6.85 per 1000 people. The results showed an increased risk of hives in children with T1DM compared to the other participants.

The results showed an increased risk of hives in children with T1DM compared to the other participants. To explain the phenomenon, the hypothesis was made that T1DM could be associated with aberrant immune responses to pancreatic β cells; the fluctuation of glucose levels would activate skin mast cells and release histamine. Children with T1DM are more likely to visit the hospital and therefore pose a possibility of reporting bias compared with the healthy population [64].

Another study conducted at Maccabi Healthcare Services in Israel collected data related to patients diagnosed with chronic urticaria between 1993 and 2010. During the study, 12,778 patients were diagnosed with chronic urticaria, of which 66.3% were female and 33.6% were male. The results of the study highlighted thyroid diseases as the most common pathologies that accompanied urticaria, 9.8% of patients were also diagnosed with hypothyroidism, and in 80.9% of patients, the pathology was diagnosed within 10 years from the diagnosis of chronic urticaria. Hyperthyroidism was less common, but the prevalence was significantly higher in the experimental group than in the control group. Antithyroid antibodies were observed in 306 of the cases of patients who associated chronic urticaria with hypothyroidism; antithyroperoxidase (ATPO) was discovered more frequently in women than in men. Another autoimmune disease diagnosed during the study was rheumatoid arthritis, with most patients (82.9%) receiving the diagnosis in the first 10 years after the development of chronic urticaria.

The probability of developing T1DM was 7.703 in patients with chronic urticaria (95%CI, 4.78–12.65; *p* < 0.005) compared to the control group. The probability for women with CU was 12.92 and for men 2.34. Most of the patients were diagnosed with T1DM in the years after the diagnosis of chronic urticaria (84.8%), with no patient diagnosed in the first 6 months after the onset of chronic urticaria [62].

In a study carried out by Hayman et al., the case of a 12-year-old boy with chronic urticaria was reported who was diagnosed with chronic autoimmune thyroiditis in routine investigations. He also developed T1DM shortly after the thyroid hormone treatment was initiated [65]. Studies show that approximately 20–30% of T1DM patients have elevated ATPO; these two conditions are most often associated with autoimmune diseases, and their etiology may include common genetic factors [66].

#### 3.6.2. Alopecia

Alopecia areata (AA) is an autoimmune disease, the second most common, after androgenic alopecia. Clinically, it is characterized by non-scaly, non-inflamed, hairless macules, and more frequent lesions on the scalp and chin, but other regions can also be affected, such as the eyelashes or eyebrows. The prevalence of the disease is 1 in 1000 and the incidence is 2% globally. Most of the patients diagnosed with this pathology are young, under 30 years old, and only approximately 20% fall into the over-40 age category [67]. From a clinical point of view, alopecia areata is classified according to severity into the following types: (1) Alopecia Areata in plaques is considered the most common form of alopecia; it is characterized by the appearance of round or oval areas with a lack of hair on the head in 90% of cases or in other parts of the body. (2) Alopecia Totala (AT) is characterized by total or almost total loss of hair from the scalp. (3) Alopecia Universalis (AU) is considered the most severe form of alopecia and differs from the other types by the total loss of hair from the entire body [68].

Alopecia often leads to a psychological burden. [69]. The first evidence of autoimmunity against hair follicles in AA involves the appearance of an inflammatory infiltrate consisting predominantly of T cells in the form of a “swarm of bees” towards the bulbar region of the hair follicle. Th1 CD4₊ lymphocytes and CD8₊ cytotoxic T lymphocytes comprise an important part of the infiltrate along with an increase in antigen-containing cells, such as Langerhan cells in the perifollicular area. It is important to note that in most cases of AA, the lymphocytic attack spares the stem cell compartment, thus allowing a possible future hair growth [70].

The interconnection between AA and other autoimmune diseases has been observed since the beginning of the 2000s when studies in Singapore involving 219 people reached the following conclusions: 60.7% of patients developed atopic dermatitis, bronchial asthma, or allergic rhinitis; 2.3% thyroid diseases; 4.1% vitiligo; 3.2% were diagnosed with diabetes; and 1.4% with Down syndrome [71]. GWA studies have discovered that the simultaneous occurrence of several autoimmune diseases could be the result of the division of some components of the immune system, some cytokine profiles, and some common loci. They also reported the risk of co-occurrence of AA and RA, celiac disease, or DM type 1 based on these common loci [72].

In 2015, Makino presented a case of a 41-year-old patient, hospitalized with hyperglycemia, who, 5 months before the presentation, showed signs of AA. After laboratory investigations, the diagnosis of type III polyglandular autoimmune syndrome (Hashimoto’s thyroiditis with T1DM with AA) was made based on the antibody assays. After the initiation of insulin therapy, the patient showed a total regeneration of the hair in approximately 2–3 months [73]. Another study carried out in Tunisia between 2012-2016 in which 204 patients diagnosed with AA were included, with the aim of gathering information in connection with the epidemiological and clinical aspects and associated comorbidities, had the following results regarding the association of AA with other autoimmune diseases: thyroid diseases 12.7%, vitiligo 1.5%, T1DM two patients, APS two cases, and lichen sclerosus and atrophic one case. In clinical forms, alopecia in plaques was the one most frequently diagnosed with a proportion of 49.5%, followed by AU with 27.5% [74].

A total of 3568 people were included in a study from Boston with the aim of evaluating the prevalence of comorbidities associated with alopecia, and among the diagnosed autoimmune diseases autoimmune thyroiditis was the most common with 14.6%, followed by DM type 1 with 11.1%, SLE with 4.3%, RA 3.9%, and psoriasis and psoriatic arthritis with 6.3% [75].

In 2015, Noso and colleagues studied the link between AA and autoimmunity against thyroid cells and anti-pancreatic islet cells, managing to highlight the relationship between these and autoimmune thyroid disease through specific antibodies; however, they could not determine a link between AA and DM [76].

#### 3.6.3. Psoriasis

Psoriasis is an inflammatory skin disease that is associated with many other medical conditions and affects over 60 million adults and children worldwide. It occurs equally in men and women, with an average age of onset of 33 years. Early presentation is often seen in women, with a bimodal onset between the ages of 16–22 and 55–60 years; it is associated with two different subtypes based on genetic and immunological characteristics: early onset before 40 years of age (75% of cases) and late onset after 40 years of age [77]. From a clinical point of view, it appears in several forms. Psoriasis vulgaris is the most common form seen in clinical practice. It is characterized by well-defined, erythematous, itchy plaques covered with silvery-white scales. Other subtypes of psoriasis are inverted psoriasis, guttate psoriasis, and pustular or erytodermic psoriasis [78]. The pathogenesis of psoriasis is multifactorial, genetics being the main contribution, especially in people with plaque psoriasis with early onset. This was reported by studies on twins, with heredity estimated at 60–90.5%. More than 60 loci of susceptibility have been identified, the pathogenesis probably being an interconnection between T cells, dendritic cells, and keratinocytes, and the IL-23/Th17 pathway being the key player of immune activation, chronic inflammation, and keratinocyte proliferation [79].

Diabetes and psoriasis are common conditions that can have catastrophic consequences. Both diseases are common comorbidities, with diabetes being a risk factor for psoriasis and vice versa. Patients with Psoriasis are more prone to obesity, cardiovascular diseases, non-alcoholic hepatitis, diabetes, and metabolic syndrome than the general population. This may be related to genetic traits, pathogenic inflammatory pathways, and common risk factors [80]. In the last decades, some studies have indicated that psoriasis belongs to an autoimmune spectrum with common loci, genetic susceptibility, molecular mechanisms, and treatment. In addition, some epidemiological studies have documented the association of psoriasis with other autoimmune diseases [81]. While some studies in the literature support this notion, other studies report contrary evidence. Such evidence would be the absence of genetic risk factors that are often shared by different autoimmune diseases such as the involvement of the PTPN22 gene, along with the absence of B-cell activation, disease-specific antibodies, or a determined autoantigen [82]. A study conducted between 2017–2019 with the aim of highlighting the association of T1DM and psoriasis in children and adolescents had the following conclusions: out of 166 subjects, 8% were diagnosed with psoriasis, with a prevalence 4 times higher than that reported in the Italian pediatric population (2.1%) [83]. In 2015, DiConstanzo and associates conducted a study in which 191 children with T1DM took part, concluding the presence of secondary autoimmune diseases in 23.6% of the participants: 27 children developed thyroid diseases, 21 had celiac disease, and 1 patient had vitiligo. Psoriasis was detected in 9 patients with a percentage of 4.7%, the ratio of girls/boys being 8:1, all with plaque psoriasis [84].

Other studies also analyzed the association of psoriasis with other autoimmune diseases and, although they discovered a predilection for associated secondary disorders, such as vitiligo, DM type, autoimmune thyroiditis, RA, or BII, they concluded that in the absence of solid evidence this subject requires more detailed research [85].

### 3.7. T1DM and Rheumatological Autoimmune Pathology

Diabetes mellitus is widely recognized for morbidity and premature mortality due to micro- and macrovascular complications, but although less known, studies demonstrate the association of this pathology with musculoskeletal conditions. Patients with diabetes can develop different symptoms or musculoskeletal syndromes, which can affect soft tissues, nerves, muscles, or tendons. While some conditions are observed in the general population, with an increased prevalence in the diabetic population, studies have shown that certain musculoskeletal conditions are specific to diabetic people [86]. High levels of insulin and tissue glucose affect many of the cells and key components of the connective tissue matrix. Although the tissue changes in DM are well documented, the clinical impact of these changes remains less studied [87].

Next, we will try to review the most important and recent advances regarding the association between Type 1 DM and rheumatic diseases.

#### 3.7.1. Rheumatoid Arthritis

Rheumatoid arthritis (RA) is one of the most common autoimmune disorders, affecting approximately 1% of the world’s population. The exact cause of RA is not known; however, disease initiation appears to result from an interaction between genetic susceptibility and environmental factors. RA is characterized by the appearance of inflammatory processes in the synovium of the joint, which ultimately leads to the destruction of the cartilaginous and bone elements of the joint [88]. The exact etiology of RA is unknown, but the existence of genetic and environmental factors associated with the disease has been demonstrated. Among the main genetic factors, DRB1 is the most important risk factor, but alleles within the HLA-DR4 group are also strongly associated with the disease [89]. Among the environmental factors, the main ones involved in increased susceptibility to RA are coffee, smoking, pollution, and exposure to silicon. Infectious factors such as EBV, mycoplasma, and proteus have been implicated in RA [90]. Rheumatoid factor RF is closely associated with RA and can confirm a clinically suspected diagnosis or characterize the severity of the disease. RF is not specific to RA, because it can be reactive for about 5% of the general population, or 15% of the elderly. Anti-citrullinated protein antibodies (ACPA) have also proven to be a useful diagnostic and staging tool. ACPA are almost as sensitive as serum FT, but are far more specific than it, with a specificity of over 90% [91]. In a patient with inflammatory arthritis, the presence of rheumatoid factor, anti-CCP antibodies, elevated C-reactive protein, or elevated ESR suggests a diagnosis of rheumatoid arthritis. The initial evaluation must also include a hematological balance associated with the complete evaluation of renal and hepatic functions [92].

Although diabetes is not recognized as an independent risk factor for the development of rheumatoid arthritis, in medical practice the two conditions are often seen to be associated. However, the role played by environmental and genetic factors in the development of autoimmunity associated with RA is not yet clear [87].

A study conducted in Sweden in which 3093 subjects took part, of which 1419 had RA, had the following conclusions: RA and T1DM are associated, this association was specific for patients with positive ACPA, and the risk of developing a T1DM in patients with RA could be partially attributed to the presence of the 620W PTPN22 allele, which may represent a common pathway in the pathogenesis of the two diagnoses [93]. Another observational study conducted in the Washington University Diabetes Center between 2011–2018, which included 1212 subjects, with a ratio of 51.8% women, reported a percentage of 6.5% systemic rheumatic diseases, and 63.3% of them were associated with other autoimmune diseases such as hypothyroidism, hyperthyroidism, pernicious anemia or celiac disease. The most common musculoskeletal condition in women was RA, with an incidence of 4.3%. Systemic lupus erythematosus and Sjogren’s disease had a percentage of 1.8% and 1%, respectively. The study indicated an increased risk of musculoskeletal diseases in women with DM1 than in men. Furthermore, it underlined the additional increased risk of other autoimmune diseases in patients with DM and rheumatic diseases [94].

#### 3.7.2. Sjogren’s Syndrome

Sjogren’s syndrome (SS) is a chronic autoimmune rheumatic illness marked by lymphocyte infiltration of the exocrine glands and other organs along with the generation of autoantibodies in the blood. This illness typically takes months or years to develop gradually and insidiously. Dry eyes, a dry mouth, exhaustion, soreness in the muscles and skeleton, and inflammation of the main salivary glands are typical symptoms. Dryness of the entire body, or Sicca syndrome, can occur in some situations as a result of the exocrine glands’ increasing deterioration and malfunction [95].

Sjogren’s syndrome is a multisystemic disorder in terms of clinical presentation, disease course, and diagnosis. As of now, there is not a single clinical, laboratory, or radiological characteristic that might be used as the “gold standard” for identifying or classifying this illness. A biopsy of the small salivary glands is the primary investigation in the diagnosis of the illness. The most significant biomarkers found to date have been incorporated into the categorization criteria for primary Sjogren’s syndrome: anti-SS-A (Ro) antibodies and anti-SS-B (La) antibodies. Rheumatoid factor RF and antinuclear antibodies ANA are other frequently tested markers in this disease. The ability of diagnostic indicators to exist years before the onset of clinical symptoms is another crucial factor. Anti-Ro and anti-La immunoglobulins have been shown to be detectable up to 18–20 years prior to the onset of symptoms and the diagnosis of Sjogren’s syndrome. [96]. The pathogenesis of SS is uncertain, just like other autoimmune illnesses. Exposure of vulnerable individuals to particular environmental factors plays a significant role in the dysregulation of the immune system and the development of the disease. More specifically, a defect in the innate immune system, particularly in the early stages of the disease, would play a crucial part in the pathogenesis of SS through a mechanism involving the interferon (IFN) pathway. On the other hand, it appears that a key factor in the SS development is the acquired immune system. The illness progresses as a result of B cells’ ongoing activation and the growth of Th1 and Th17 cells [97].

H. Shimomura and associates report a rare case of autoimmune polyglandular syndrome type III of a 57-year-old woman, known to have DM type 2, who was presented for the treatment of a lip tumor, hand ulcers, and alopecia. Following the tests performed, she was diagnosed with type 1 DM, with positive GADA and ICA, Graves’ disease, SS, SLE, alopecia, autoimmune neutropenia, and squamous cell carcinoma [98].

#### 3.7.3. Systemic Lupus Erythematosus

Systemic lupus erythematosus (SLE) is a chronic inflammatory disease with multisystemic damage and is marked by a variety of clinical manifestations; the main ones involved being inflammatory skin lesions, arthritis, pleurisy, pericarditis, and renal manifestations [99]. Recent data and studies published all over the world have allowed more accurate estimates of the incidence and prevalence of SLE. The estimated incidence of 23.2 cases per 100,000 people in North America is the highest in the world. Lupus occurs more frequently in African Americans, Hispanics, and Asians than in Caucasians [100]. Because the symptoms and indications of lupus vary, diagnosing it can be challenging. Different manifestations, such as hemolytic anemia, convulsions, or psychosis, alone or in combination, can be ascribed to other causes or illnesses, despite the fact that symptoms like arthritis and the typical malar rash are clearly identifiable. In addition, the absence of a validated test makes diagnosis more difficult. The results of an antinuclear antibody test are a diagnostic indicator, but they must be interpreted in light of other clinical characteristics, such as the history, physical exam, and other tests [101].

The generation of autoantibodies against a variety of nuclear antigens, which is a distinctive immunological characteristic of SLE patients and reflects the underlying loss of self-tolerance, is sufficient but not required for the illness to develop. Years before the onset of an obvious clinical condition, detectable autoantibodies may emerge. Since virtually all SLE patients have a large level of ANA, this test is incredibly sensitive. Anti-nuclear antibodies are also implicated in other autoimmune disorders; therefore, the specificity is limited. Antibodies such as anti-Ro (25–40%), anti-La (10–20%), and anti-Sm (10–30%) are positive in SLE. Anti-Ro and anti-La antibodies are crucial for SLE patients planning pregnancies since they are linked to congenital heart block and neonatal lupus in the fetus. The most specific SLE antibody, anti-Sm, can be used as a diagnostic sign when positive. The specificity of anti-dcDNA varies depending on the disease’s activity, especially when it manifests in high titers. Components of C3 and C4 are frequently found in low amounts. Anti-phospholipid antibodies are found in 29–46% of SLE patients, and up to 50% of them may have pregnancy morbidity or arterial or venous thrombosis. [102]. The condition has a hereditary component, with 24–35 percent concordance in monozygotic twins and 2–5% in dizygotic twins. HLA-DRB1∗1501 and HLA-DRB1∗0301 are linked to a 2–3 times greater incidence of SLE in the Caucasian population [99].

The most prevalent autoimmune illnesses linked to diabetes, according to a recent study on the proportion of T1DM patients who have them, are thyroid disorders (20.1%), rheumatic disorders (3.4%), and BII (1.4%), with RA being the most common diagnosis. In terms of hypothyroidism, celiac disease, and Sjogren’s SLE, women were shown to have the majority of these autoimmune disorders [103]. Due to the quantity of immune complexes, autoantibodies, the relationship with HLA, and the accumulation of lymphocytes, monocytes, and macrophages at the level of the lesions, it is thought that RA and SLE are rheumatic illnesses that have an immunological origin. The discovery that the PTPN 1858T variant is linked not just to diabetes but also to RA, SLE, and other autoimmune disorders supports the hypothesis that a number of autoimmune diseases may share certain common genes or susceptibility pathways. Another piece of evidence that supports the aforementioned concept is the role of CTLA4 in the susceptibility to Graves’ disease, autoimmune hypothyroidism, DM type 1, and SLE [104]. Table 1 summarizes the autoimmune diseases and corresponding antibodies.

## 4. Moving towards Personalized Patient-Centered Management

**Figure 1:**
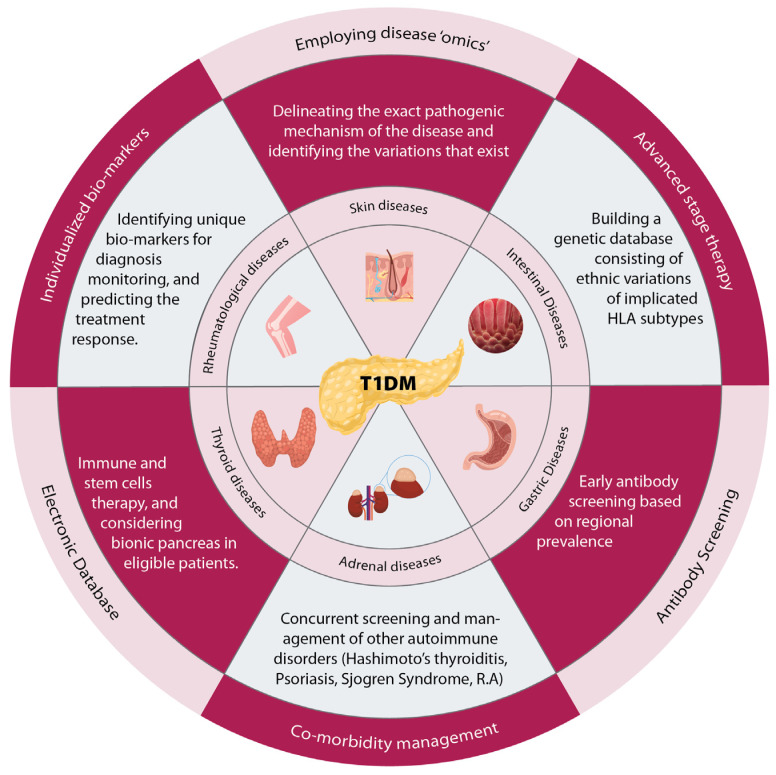
Personalized Medicine as an approach to T1DM and associated autoimmune diseases. T1DM is associated with multiple other endocrine abnormalities—including thyroid, adrenal, gastrointestinal, rheumatological, and dermatological—that might be inapparent initially. Suggested approaches for managing these conditions are based on maintaining an electronic database with a recording of antibody screening and identifying biomarkers.

Over the last decad the management of autoimmune diseases including T1DM has seen a significant shift from a blanket approach to a personalized approach (Table 2) [105,106,107,108,109,110,111,112]. Autoimmune diseases are complex conditions that are brought on by a combination of hereditary and environmental causes, and are often present with associations with other autoimmune diseases, contributing to adding mortality and morbidity. In the case of these disorders, a variety of therapeutic strategies may be utilized to bring about remission or, at the least, to lessen the severity of the symptoms [113]. Precision medicine, also known as customized or personalized medicine, has recently acquired popularity in the scientific, medical, and public realms and is regularly touted as the future paradigm of healthcare delivery. Its purpose is to combine patient-specific information in order to personalize care and get the best potential outcome. Although precision medicine is not yet fully established, its application is increasingly permeating clinical practice, and the goal of individualizing patient treatment is now closer to being achieved. In view of our understanding of associated autoimmune disorder in T1DM, we have proposed several management strategies to personalize the therapy. These strategies can lead to an adoption of a more focused clinical approach to these patients as well as enhance the effectiveness of the ongoing treatment (Figure 1).

## 5. Conclusions

T1DM is an autoimmune disease, and the detection of autoimmunity markers is clear evidence in the favor of this mechanism. Over the years, studies have described frequent associations between T1DM and various autoimmune diseases that affect multiple systems such as the endocrine, digestive, skin, and musculoskeletal system. Genetic and environmental factors are involved in the emergence of these associations, but their exact role is not yet clear. The coexistence of T1DM and another autoimmune disease in the same patient has a major impact on the patient’s quality of life, affecting glucose metabolism, preventing effective insulin therapy, and influencing diabetes control. Hence, we recommend personalizing the treatment of patients with diabetes mellitus in line with their comorbidities and the course of the disease. We also highlight the need for making antibody screening a regular part of disease protocol at least in regions where autoimmune diseases are prevalent, if not everywhere. The co-existent autoimmune diseases thus treated in tandem can help better patient outcomes. Future studies are warranted to contribute region-wise prevalence data of these associations as regional and ethnic variations have been largely seen; this data can thus help improve clinical decision making.

## Figures and Tables

**Table 1:** The autoimmune diseases and corresponding antibodies [4,8,11,12,27,32,49,50,52,56,76,82,92,99,102].

Autoimmune Pathology	Antibodies	Reference
Hashimoto’s thyroiditis	Antithyroid peroxidase antibodies Antithyroglobulin antibodies	[4,8]
Basedow-Graves-disease	Antithyrotropin receptor antibodies (TRAb)	[11,12]
Addison’s disease	Antibodies against 21-hydroxylase	[27,32]
Hypoparathyroidism	Antibodies against CaSR(Calcium-Sensing Receptor) Anti NALP5-antibodies	[49]
Celiac disease	Antibodies tissue transglutaminase Anti-endomysium antibodies Antigliadin antibodies	[50]
Autoimmune gastritis	Antibodies against parietal cells Antibodies against intrinsec factor	[52]
Rheumatoid arthritis	Rheumatoid factor FR AAN antinuclear antibodies	[56,76,82]
Sjogrens disease	Antinuclear antibodies AAN Anti SS-A/anti-Ro antibodies Anti SS-B/anti-La-antibodies	[92,99]
	Antinuclear-AAN-antiboies Anti SS-A/anti-Ro antibodies- Anti SS-B/anti-La antibodies Anti-Sm (Smith)-antibodies Antibodies against double-stranded DNA (anti-dc-DNAantibody)	[102]

**Table 2:** Personalized medicine and T1DM [105,106,107,108,109,110,111,112].

Personalized Medicine	Application in T1DM	References
**Personalized Diagnosis**		
**1. HbA1c in Diagnosis and Monitoring**	HbA1c is affected by glucose levels, hemoglobin, and red cell stability. Unexpected HbA1c variations can be discovered by blood investigations and genetic testing, hence allowing an individualized approach.	[105]
**2. Subcategories in T1DM**	Etiological types of T1DM can be established on basis of age of onset of diabetes and type of islet cell antibody present (GAD, ICA512/IA-2, insulin, zinc transporter 8 [ZnT8])	[106]
**3. Early diagnosis of diabetic neuropathy—tight control of DM**	Insulin and C-peptide deficiencies cause acute metabolic irregularities, persistent gene regulatory perturbations, poor neurotrophism, protein–protein interactions, and particular degenerative diseases in type 1 DPN (Diabetic Peripheral neuropathy). Hence, complications can be prevented by an individualized approach.	[107]
**Personalized Therapy**		
**1. Intensive insulin therapy**	Individually tailored insulin therapy based on HbA1c has proven to be more beneficial.	[108]
**2. Immune therapy**	Auto-aggressive T cell repertoire varies between patients and the number of residual beta-cells will directly affect insulin production post-treatment. Thus, a combination therapy can be more useful.	[109]
**3. Gene therapy**	Restoration of insulin responsiveness, suppression of autoimmunity, functional replacement of pancreatic islets, and correction of vascular and nerve damage associated with prolonged hyperglycemia can all be done with gene therapy, and the future holds great promise for the same.	[110]
**Personalized Prevention and Risk Stratification**		
**1. Primary prevention of T1DM in pre-stage 1**	Individual genotyping of the genes on which more than 30% of the T1DM genetic risk is attributed to human leukocyte antigen (*HLA*) complex genes and more than 50 non-*HLA* loci.	[111]
**2. T1D genetic risk scores (T1D-GRS)**	High T1D-GRS has low PPV in populations with low prevalence.	[111,112]

## Data Availability

All the data are available in this manuscript.
